# Hybrid Nanosystem Formed by DOX-Loaded Liposomes and Extracellular Vesicles from MDA-MB-231 Is Effective against Breast Cancer Cells with Different Molecular Profiles

**DOI:** 10.3390/pharmaceutics16060739

**Published:** 2024-05-30

**Authors:** Luiza Marques Paschoal Barbosa, Eliza Rocha Gomes, André Luis Branco de Barros, Geovanni Dantas Cassali, Andréa Teixeira de Carvalho, Juliana de Oliveira Silva, Ana Luiza Pádua, Mônica Cristina Oliveira

**Affiliations:** 1 Department of Pharmaceutical Products, Faculty of Pharmacy, Universidade Federal de Minas Gerais, Av. Antônio Carlos, 6627, Belo Horizonte 31270-901, MG, Brazil; elizarochagomes@gmail.com (E.R.G.);; 2 Institute of Regenerative Medicine and Biotherapies (IRMB), Hôpital Saint-Eloi, 34295 Montpellier, France; 3 Department of Clinical and Toxicological Analysis, Faculty of Pharmacy, Universidade Federal de Minas Gerais, Av. Antônio Carlos, 6627, Belo Horizonte 31270-901, MG, Brazil; 4 Department of General Pathology, Institute of Biological Sciences, Universidade Federal de Minas Gerais, Av. Antônio Carlos, 6627, Belo Horizonte 31270-901, MG, Brazil; 5 Instituto René Rachou, Fiocruz Minas, Av. Augusto de Lima, 1715, Barro Preto, Belo Horizonte 30190-002, MG, Brazil

**Keywords:** extracellular vesicles, hybrid nanosystems, doxorubicin, breast cancer, liposomes

## Abstract

Drug delivery selectivity is a challenge for cancer treatment. A hybrid pegylated pH-sensitive liposome–extracellular vesicle isolated from human breast cancer cell MDA-MB-231 was developed to investigate its in vitro activity against breast cancer cells of different molecular profiles to overcome this inconvenience. The hybrid nanosystem was produced by film hydration, and doxorubicin (DOX) was encapsulated in this system using the ammonium sulfate gradient method. The characterization of this hybrid nanosystem revealed a mean diameter of 140.20 ± 2.70 nm, a polydispersity index of 0.102 ± 0.033, an encapsulation efficiency of doxorubicin of 88.9% ± 2.4, and a great storage stability for 90 days at 4 °C. The fusion of extracellular vesicles with liposomes was confirmed by nanoflow cytometry using PE-conjugated human anti-CD63. This hybrid nanosystem demonstrated cytotoxicity against human breast cancer cell lines with different molecular subtypes, enhanced anti-migration properties, and exhibited similar cellular uptake to the free DOX treatment. Preliminary acute toxicity assessments using Balb/C female mice indicated a median lethal dose of 15–17.5 mg/kg, with no evidence of splenic, liver, heart, bone marrow, and renal damage at a dose of 15 mg/kg. These findings suggest the hybrid formulation as a versatile nanocarrier for the treatment of various breast cancer subtypes.

## 1. Introduction

**Figure 1:**
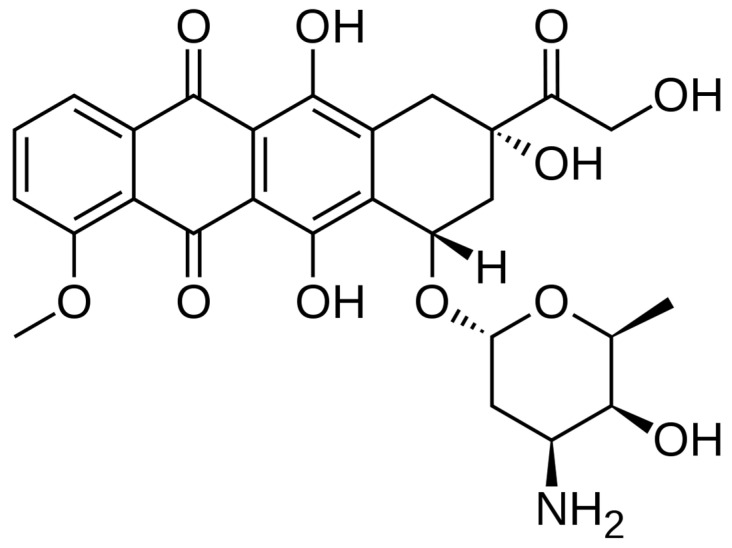
Doxorubicin (DOX) molecular scheme.

Cancer remains a major challenge for public health, with an estimated 9.7 million deaths in 2022. Breast cancer is the second most common diagnostic type of cancer in women, with 11.5% (2.29 million) of new diagnoses in 2022 [1]. Despite therapeutic advancements, treatments often lack selectivity and the recent reports on anthracycline resistance pose an issue, requiring innovative strategies for targeted drug delivery while minimizing systemic toxicity [2,3,4]. Doxorubicin (DOX), represented in Figure 1, is an anthracycline used as a chemotherapeutical drug to treat several cancer types, including breast cancer [5]. However, the clinical use of DOX requires attention to its side effects, particularly its dose-dependent cardiotoxicity [6]. In order to avoid these side effects, new formulations with reduced toxicity and increased efficacy to improve patient treatment and comfort are necessary. Many nanostructures are being explored as drug delivery systems to address these challenges, and liposome-encapsulating DOX is a significant part of the nanosystems in clinical trials or already in use. Despite its clinical use, these nanocarriers did not show improvements regarding therapeutic efficacy compared to the conventional DOX treatment [5,7]. Fusing extracellular vesicles (EVs) and liposomes emerges as an intriguing alternative for enhancing DOX’s selectivity, uptake, and therapeutic effectiveness. Evs are a class of lipid bilayer particles secreted by various cell types, and can carry surface receptors, proteins, nucleic acids, and other substances [8]. Their role in cell communication may increase drug accumulation in tumor tissue, and improve treatment efficacy, which makes Evs an interesting option for active drug delivery [8,9]. Furthermore, regarding Evs encapsulating DOX, some studies showed that these drug delivery systems could decrease DOX cardiotoxicity [9]. We fused pegylated pH-sensitive liposomes with Evs isolated from the MDA-MB-231 human breast cancer cell line (HF-pHSL/EV-DOX) to assess the viability of a hybrid nanosystem. We investigated the cytotoxic activity, and its cellular uptake and migratory activity in human breast cancer cell lines with different molecular subtypes (MDA-MB-231, MCF-7, SKBR-3). We have preliminarily performed an investigation of the acute toxicological profile of HF-pHSL/EV-DOX in Balb/c female mice.

## 2. Materials and Methods

### 2.1. Chemicals

1,2-Dioleoyl-sn-glycero-3-phosphoethanolamine (DOPE) and 1,2-distearoyl-sn-glycero-3-phosphoethanolamine-N-[amino(polyethyleneglycol)-2000 (DSPE-PEG2000) were supplied by Lipoid GmbH (Ludwigshafen, Germany). Cholesterol hemisuccinate (CHEMS), sodium hydroxide, phosphate-buffered saline (PBS), and 4-(2-hydroxyethyl)piperazine-1-ethanesulfonic acid (HEPES) were obtained from Sigma-Aldrich (St. Louis, MO, USA). DOX was generously provided by Eurofarma (São Paulo, Brazil). A total exosome isolation reagent kit was obtained from Thermo Fisher Scientific (Waltham, MA, USA). PE anti-human CD63 monoclonal antibody was obtained from BioAlbra Biotecnologia (Viçosa, Brazil).

### 2.2. Cells

The MDA-MB-231 (triple negative), MCF7 (ER+/PR+/HER2+), and SKBR3 (HER2+) human breast cancer cells were purchased from the American Type Culture Collection (ATCC) (Manassas, VA, USA). Dulbecco’s Modified Eagle’s Medium (DMEM), Minimum Essential Medium Eagle (MEM), McCoy, trypsin, tris(hydroxymethyl)aminomethane (Tris base), human recombinant insulin, and Sulforhodamine B (SRB) were obtained from Sigma-Aldrich (St. Louis, MO, USA). Fetal bovine serum (FBS) was obtained from Gibco Life Technologies (Carlsbad, CA, USA). Prior to the experiments, all cell lines were screened for mycoplasma by polymerase chain reaction (PCR), with negative results.

### 2.3. Isolation of Extracellular Vesicles

MDA-MB-231 breast cancer cells were grown in DMEM medium supplemented with 10% of ultracentrifuged FBS, and maintained at 37 °C and 5% CO_2_ in a humidified atmosphere. The cell supernatant was removed from the cell culture flask T-75 when the cells reached an approximate confluence of 80%. The total exosome isolation reagent was added to the cell supernatant (1:2 *v*/*v* ratio, respectively) and maintained at 2 to 8 °C for 15 h. Then, the mixture was centrifuged at 10,000× *g*, for 1 h, at 4 °C, using the Thermo Scientific centrifuge, with model Heraeus Multifuge X1R (Waltham, MA, USA). The EVs’ pellet obtained was resuspended in HBS for analysis or in a mixture of methanol and chloroform (1:1 *v*/*v* ratio) for preparation of the hybrid nanosystem.

### 2.4. Preparation of HF-pHSL/EV-DOX

HF-pHSL/EV-DOX was prepared by the Bangham method, as described by Gomes and coworkers [10]. Lipids were solubilized in chloroform and added to a round-bottom flask. For each mL of liposome, 7.99 × 10^10^ particles of EVs per mL were added. EVs dissolved in methanol:chloroform (1:1 *v*/*v*) were added to solubilized DOPE, CHEMS and DSPE-PEG_2000_ (total lipid concentration 20 mM, molar ratio of 5.7:3.8:0.5, respectively) in a round-bottom flask. A lipid film was obtained by evaporating the solvents under reduced pressure. Next, CHEMS molecules were ionized with a NaOH 0.228 M solution and an ammonium sulfate solution (300 mM, pH 7.4) was used for lipid film hydration. The size of the hybrid vesicles was calibrated by the extrusion method in the Lipex Biomembranes extruder using polycarbonate membranes of 0.4 µm, 0.2 µm and 0.1 µm (model T001, Vancouver, Canada) [11]. The hybrid formulation was submitted to dialysis against HBS to remove external ammonium sulfate and then the dispersion was incubated with a DOX solution (2 mg/mL) for 2 h at room temperature. Non-encapsulated DOX was removed from the dispersion with another dialysis against HBS. Blank breast-tumor-derived EVs fused with pegylated pH-sensitive liposomes (HF-pHSL/EV) and pegylated pH-sensitive liposomes containing DOX (pHSL-DOX) were prepared in the same way without the addition of DOX and EVs, respectively.

### 2.5. HF-pHSL/EV-DOX Characterization

#### 2.5.1. Determination of the Diameter, Polydispersity Index, Zeta Potential, and Particle Concentration

The mean diameter and polydispersity index (PDI) of HF-pHSL/EV-DOX were determined by dynamic light scattering (DLS). The zeta potential was evaluated by DLS associated with electrophoretic mobility. To perform both analyses, 50 μL of HF-pHSL/EV-DOX was diluted in 1 mL of HBS, and the Zetasizer Nano ZS90 equipment was used (Malvern Panalytical Ltd., Malvern, UK).

The particle concentration was measured by nanoparticle-tracking analysis (NTA) using the NanoSight NS300 (Malvern Panalytical Ltd., Malvern, UK). Samples were diluted in filtered HBS buffer and manually introduced into the equipment, evaluated 5 times, capturing 60 s videos at 25 °C. The NTA 3.3 software was used to perform the analysis of particle concentration.

#### 2.5.2. Determination of DOX Concentration

The DOX content was measured by high-performance liquid chromatography (HPLC) with a mobile phase composed of methanol and phosphate buffer at pH 3.0 (65:35 v/v). Samples were injected at a volume of 20 μL, and separation was performed with an ACE^®^ C8 column, 25 cm × 4.6 mm, 5 μm (Merck, Darmstadt, Germany) at a flow rate of 1.0 mL/min. DOX detection was performed in 2475 fluorescence mode (Waters Instruments, Milford, MA, USA) with excitation and emission wavelengths of 470 nm and 555 nm, respectively [10]. The HF-pHSL/EV-DOX vesicles were opened with isopropyl alcohol (1:2 *v*/*v*, respectively) and diluted in the mobile phase. The encapsulation percentage (EP) of DOX in HF-pHSL/EV-DOX was calculated according to Equation (1):

(1) $$\mathrm{DOX}\;\mathrm{encapsulation}\;\mathrm{percentage}\;\%=\frac{\left[\mathrm{DOX}\right]\;\mathrm{in}\;\mathrm{purified}\;\mathrm{hybrid}}{\left[\mathrm{DOX}\right]\;\mathrm{total}\;\mathrm{amount}\;\mathrm{incubated}}\times 100$$

#### 2.5.3. Determination of Protein Concentration

The protein concentration EVs isolated from MDA-MB-231 cell culture, blank HF-pHSL/EV, and blank pHSL was determined using the Pierce BCA Protein Assay Kit (Thermo Fisher Scientific, Waltham, MA, USA). Samples were added to 96-well plates and diluted with the working reagent. The plates were then incubated at 37 °C for 30 min, and absorbance at 562 nm was measured using the SpectraMax Plus 384 (Molecular Devices^®^, San Jose, CA, USA). The results were analyzed using SoftMax Pro 6.51^®^ software.

#### 2.5.4. Nanoflow Cytometry

Briefly, 25 μL of the samples (EVs isolated from MDA-MB-231 cell culture, blank HF-pHSL/EV, and blank pHSL) was incubated with 10 μL of a 1:5 solution of PE-conjugated anti-CD63 monoclonal antibody (BioAlbra Biotecnologia, Viçosa, Brazil) in HBS buffer (*v*/*v*, respectively), and 25 μL of HBS for 30 min at room temperature in the dark. The total volume amount was made up to 250 μL using HBS buffer. Analysis was performed using a flow rate setting of 30 μL/min, and the sample acquisition time was 2 min/sample. Data analysis was performed by FlowJo software v.10.x (FlowJo, LLC., Vancouver, BC, Canada).

### 2.6. Storage Stability

The storage stability of HF-pHSL/EV-DOX was determined for 120 days. The formulation was maintained at 4 °C in HBS buffer and aliquots were collected after 0, 7, 14, 30, 60, 90, and 120 days of preparation. The hybrid was characterized for its chemical and physicochemical parameters such as mean diameter, PDI, zeta potential, and DOX EP, as mentioned in 2.5.1 and 2.5.2. To separate the hybrid from the aqueous phase containing released DOX, the aliquots were submitted to ultrafiltration using centrifugal devices Amicon^®^ Ultra—50 kDa (Merck S.A., Darmstadt, Germany) pre-treated with Tween 20 5% (*v*/*v*) solution for 24 h [12]. The released DOX (aqueous phase) and HF-pHSL/EV-DOX after ultrafiltration (purified hybrid) were collected, diluted, and the concentration of DOX was measured by HPLC as described above. The EP was calculated as in Equation (1) and the measurements were performed in triplicate.

### 2.7. Release Study

The pH sensitivity of HF-pHSL/EV-DOX was assessed by dialysis in HBS-saline buffer using cellulose membranes with a cutoff size of 12 kDa (Sigma-Aldrich, St. Louis, USA). Dialysis membranes were filled with 250 μL of HF-pHSL/EV-DOX and incubated in 100 mL of HBS saline buffer at different pH values, to simulate DOX release in normal tissue and acidic environment of the tumor, at pH 7.4 and 5.0, respectively. The flasks were kept under agitation (156 rpm) at 37 °C in an orbital shaker (IKA KS 4000i, Campinas, Brazil). At 1 h, 4 h, 12 h and 24 h, aliquots were collected and DOX concentration inside the dialysis bag was quantified according to the HPLC method described above.

### 2.8. Cell Culture

Human breast tumor cells MDA-MB-231 (triple negative), MCF7 (ER+/PR+/HER2+), and SKBR3 (HER2+) were cultured in DMEM, MEM (supplemented with 10 mg/L insulin), and McCoy media, respectively, and all supplemented with 10% FBS. Cell lines were cultivated with penicillin (100 IU/mL) and streptomycin (100 μg/mL) and maintained at 37 °C and 5% CO_2_ in a humidified atmosphere. Prior to the experiments, all cell lines were screened for mycoplasma by polymerase chain reaction (PCR), with negative results.

#### 2.8.1. Sulforhodamine B Assay

MDA-MB-231 cells were plated at a density of 6 × 10^3^ cells/well in 96-well plates and incubated for 24 h, at 37 °C and 5% CO_2_. MCF-7 and SKBR-3 cells were plated at a density of 1 × 10^4^ cells/well in 96-well plates and incubated for 24 h, at 37 °C and 5% CO_2_. After the incubation time, the proposed treatments [free DOX, DOX-loaded pegylated pH-sensitive liposomes (pHSL-DOX), HF-pHSL/EV-DOX, or blank HF-pHSL/EV] were added to the wells. After incubation time (48 h), 100 μL of 10% trichloroacetic acid (TCA) was added and incubated for 1 h at 4 °C. The plates were washed with water four times after the fixation period. Then, 100 μL of sulforhodamine B was added and incubated for 30 min. After the incubation period, the plates were washed four times with 1% (*v*/*v*) acetic acid. Finally, 100 μL of 10 mM Tris-based solution (pH 10.5) was added to solubilize the dye that had bound to the proteins. The absorbance was determined at 510 nm using the spectrophotometer SpectraMax Plus 384 (Molecular Devices, San Jose, CA, USA). The IC_50_ values were calculated using GraphPad Prism 9.0 (GraphPad Software, La Jolla, CA, USA).

#### 2.8.2. Cellular Uptake Study

The cellular uptake of DOX from HF-pHSL/EV-DOX was quantified by HPLC. MDA-MB-231, MCF-7, and SKBR-3 tumor cells were plated in triplicate in 12-well plates (5 × 10^5^ cells/well) and incubated for 24 h. Cells were treated with 1 mL of fresh DMEM containing 5 μM of free DOX, pHSL-DOX, or HF-pHSL/EV-DOX for 1 h, 4 h, and 24 h. After treatment, the cells were removed from the plates, washed with PBS three times to remove the non-internalized drug, and centrifuged (500× *g*, 5 min). Pellets were resuspended in 1 mL of isopropyl alcohol:methanol (1:1 *v*/*v*), and the samples were transferred to an ultrasonic bath to promote the cell lysis and DOX extraction. The lysed cells were centrifuged at 500× *g* for 15 min, and an aliquot of the supernatant was used to quantify intracellular DOX by HPLC as described in Section 2.5.2. The DOX cellular uptake was calculated using Equation (2).

(2) $$\mathrm{DOX}\;\mathrm{cellular}\;\mathrm{uptake}\;\mathrm{percentage}\;\%=\frac{\left[\mathrm{DOX}\right]\;\mathrm{internalized}\;\mathrm{in}\;\mathrm{cells}}{\left[\mathrm{DOX}\right]\;\mathrm{total}}\times 100$$

#### 2.8.3. Migration Test—Wound-Healing Assay

MDA-MB-231, MCF-7 and SKBR-3 cells were plated in triplicate at a density of 2.0 × 10^5^ cells/well in 24-well plates and incubated at 37 °C for 24 h. A straight wound was made into the wells with a 10 μL pipette tip, and this “time zero area” was imaged using an AxioVert 25 microscope with an Axio Cam MRC camera attached (Zeiss, Oberkochen, Germany). After image acquisition, the wells were treated with free DOX, pHSL-DOX, DOXOPEG^®^, HF-pHSL/EV-DOX, or blank HF-pHSL/EV in 1 mL of medium with 1% FBS, at a DOX concentration of 500 nM for each treatment. After 24 h of incubation at 37 °C, the cells were fixed with 4% formaldehyde for 10 min. Images along the treated wounds were also obtained in phase contrast to obtain the “time 24 h area”. The areas of all wounds were obtained using the MRI Wound Healing Tool plugin for the free version of the Image J 1.45 software (National Institutes of Health, Bethesda, CA, USA). The wound-healing percentage was calculated using the following Equation (3):

(3) $$\text{Wound-healing}\;\mathrm{percentage}\;\%=100-\left(\frac{\mathrm{wound}\;\mathrm{area}\;\text{“}\mathrm{time}\;24\;h\;\mathrm{area}\text{”}}{\mathrm{wound}\;\mathrm{area}\;\text{“}\mathrm{time}\;\mathrm{zero}\;\mathrm{area}\text{”}}\right)\times 100$$

### 2.9. Acute Toxicity

Acute toxicity evaluation followed the OECD Guideline for Testing of Chemicals 423 (2001) adapted to intravenous drugs, as previously reported by our research group [13]. Acute toxicity testing is most often performed using mice as a rodent species [14]. Balb/c female mice were chosen, a standard and well-stablished model used in toxicity studies [10,15]. According to the OECD 423 guideline, females are used because they are slightly more sensitive [13]. Healthy female Balb/c mice, aged 6–7 weeks and weighing approximately 18 g, were obtained from the Centro de Bioterismo do Instituto de Ciências Biológicas da Universidade Federal de Minas Gerais (CEBIO/UFMG, Belo Horizonte, Brazil). The in vivo study was previously approved by the local ethics committee for animal use (CEUA/UFMG—Protocol n° 265/19).

The animals were divided into four groups. Each group received a single intravenous dose of HBS (control), free DOX, HF-pHSL/EV, or HF-pHSL/EV-DOX at different concentrations. The mice were monitored for 14 days in terms of their behavior, weight, and mortality. The final weight variation was calculated by the difference between the final and initial weight of the animals. After that, the animals were intraperitoneally anesthetized with a mixture of xylazine (15 mg/kg) and ketamine (80 mg/kg). The blood was collected by puncture of the brachial plexus for hematological and biochemical analyses, and the organs were collected for histopathological analyses.

**Figure A1:**
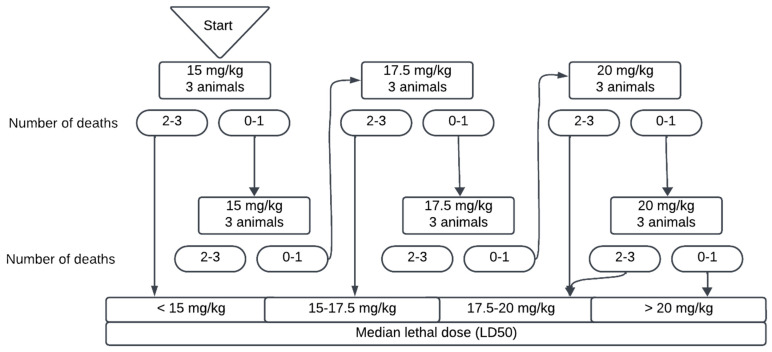
Treatment scheme used to assess the median lethal dose (LD50) after treatment with free DOX.

**Figure A2:**
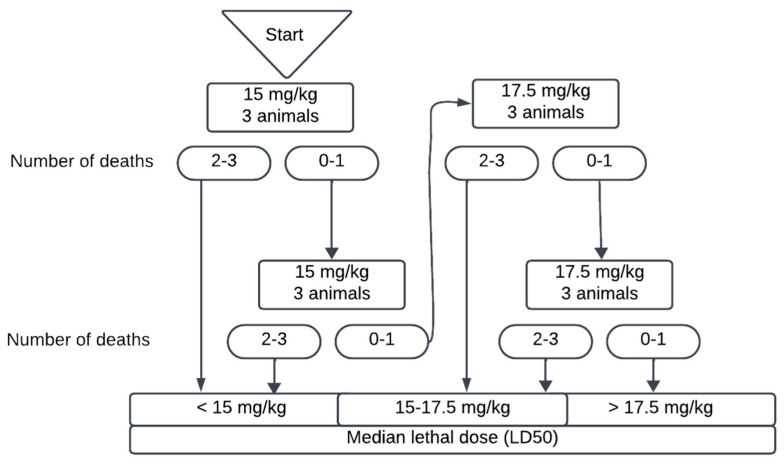
Treatment scheme used to assess the median lethal dose (LD50) after treatment with HF-pHSL/EV-DOX.

In previous studies conducted by our research group, the median lethal dose (LD50) obtained ranged between 12.5 mg/kg and 15 mg/kg for treatment with free DOX, while for treatments with pHSL-DOX, the LD50 ranged between 17.5 mg/kg and 20 mg/kg [10]. Based on these findings, the initial dose selected for treatment with free DOX and HF-pHSL/EV-DOX was set at 15 mg/kg. Following the recommendation of the Organization for Economic Co-operation and Development [13], each treatment group initially comprised three animals. If the tested dose resulted in mortality among 2 or more animals within the group, testing on an additional 3 animals at the next lower dose level would be required. If the tested dose caused mortality in 1 or no animals, the next step would involve testing an additional 3 animals at the same dose level. If the outcome of 1 or no mortality persists, further testing on 3 animals with a higher dose would be necessary. The dose scheme used to assess the median lethal dose (LD50) after treatments with free DOX and HF-pHSL/EV-DOX is presented in Appendix A (Figure A1 and Figure A2, respectively).

#### 2.9.1. Hematology and Biochemistry Analyses

Hematological analyses were conducted with the quantification of parameters including hemoglobin concentration, red blood cell count, hematocrit, total white blood cells, granulocytes, agranulocytes, red cell distribution width, and platelets. The HEMOVET 2300 analyzer (Hemovet, São Paulo, Brazil) was used to assess these parameters.

For biochemical analyses, plasma was obtained with a centrifugation at 3000 rpm for 15 min, and stored at −20 °C if needed. Biochemical tests were conducted via spectrophotometric analysis using the semi-automatic analyzer model Bioplus BIO-2000 (São Paulo, Brazil), with commercial kits (Labtest, Lagoa Santa, Brazil). Hepatic function was assessed through alanine aminotransferase (ALT) and aspartate aminotransferase (AST) assays, and renal activity was determined via urea and creatinine concentration measurement, while cardiac function was evaluated through creatine kinase isoform MB (CK-MB) quantification.

#### 2.9.2. Histopathological Analysis

Histopathological analysis was performed on liver, kidneys, spleen, bone marrow, and heart. Tissue samples were fixed in 10% formalin buffer for 24–48 h, dehydrated in alcohol, and incorporated in paraffin blocks. Sections of 4 µm thickness were obtained and stained with hematoxylin and eosin (H&E). These sections were evaluated by trained pathologists, and images were captured using a camera attached to an optical microscope (Olympus BX-40; Olympus, Tokyo, Japan).

### 2.10. Statistical Analyses

Statistical analyses were performed using the GraphPad Prism software (version 9.0.2, La Jolla, CA, USA). Normality and homoscedasticity of variance were tested by D’Agostino and Shapiro–Wilk tests, respectively. The comparison between the experimental groups was made by analysis of variance (one-way ANOVA followed by Tukey’s test). For statistical analyses, differences were considered statistically significant when *p* < 0.05.

## 3. Results

### 3.1. Characterization of HF-pHSL/EV-DOX

The hybrid composed of pegylated pH-sensitive liposome/EV isolated from the human MDA-MB-231 breast cancer cell line exhibited an average diameter of approximately 140.2 ± 2.7 nm (Table 1). HF-pHSL/EV-DOX displayed a polydispersity index (PDI) value of 0.102 ± 0.033, and a zeta potential (ZP) close to neutrality, indicating the suitability of this monodisperse nanosystem for in vivo administration. The EP of HF-pHSL/EV-DOX was great, at 88.9 ± 2.4%. This value compares to those obtained in other studies conducted by our research group [10,11,12].

The particle concentration for the isolated EVs was 7.99 ± 0.50 × 10^10^ particles/mL, indicating high isolation yield obtained through the PEG precipitation method employing the total exosome isolation kit (ThermoFisher, Waltham, MA, USA). The hybrid formulation exhibited a higher particle concentration per mL, 5.01 ± 0.75 × 10^13^ particles/mL for HF-pHSL/EV-DOX (Table 1).

### 3.2. Protein Content of HF-pHSL/EV

The total protein content in EVs and hybrid formulations was determined using the BCA method, which involves forming a Cu^2+^-protein complex, resulting in a color change from green to purple proportional to the protein concentration. The results are shown in Table 2, which indicates that the protein concentration in blank pHSL was lower than the concentration of proteins in blank HF-pHSL/EV and EVs MDA-MB-231, suggesting the presence of EVs in the hybrid formulation.

### 3.3. Nanoflow Cytometry

**Figure 2:**
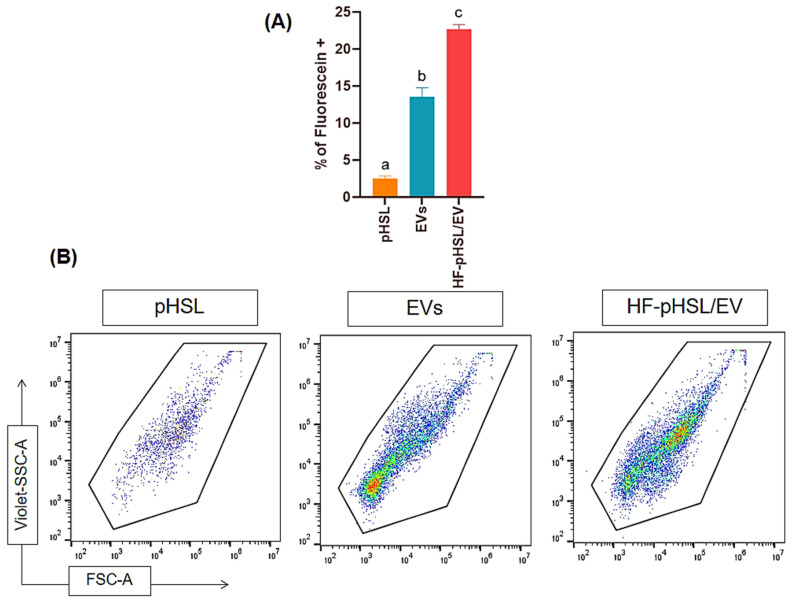
Extracellular vesicles (EV) isolation and fusion with pegylated and pH-sensitive liposomes were assessed using nanoflow cytometry. (**A**) The bar plot illustrates the mean percentages of positive fluorescent nanoparticles for all experimental groups: orange represents pegylated pH-sensitive liposomes (pHSL), blue represents EVs, and red represents the hybrid formulation HF-pHSL/EV. Different letters indicate statistically significant differences between the evaluated parameters (*p* < 0.05). (**B**) Representative density plots showing FSC-A vs. Violet-SSC-A parameters are provided for each group.

The identification of CD63 protein on the surface of the hybrid formulation was analyzed using nanoflow cytometry to confirm the fusion between liposomal vesicles and EVs. The data suggested an increased percentage of nanoparticles positive for CD63 in HF-pHSL-/EV compared to pHSL (Figure 2), evidencing the presence of these proteins on the hybrid surface.

### 3.4. Storage Stability

The storage stability of HF-pHSL/EV-DOX was determined by monitoring its chemical and physicochemical integrity for 120 days. Parameters such as mean diameter, polydispersity index (PDI), zeta potential (PZ), and concentration of the bioactive substance were evaluated to assess the stability of the nanosystem.

**Figure 3:**
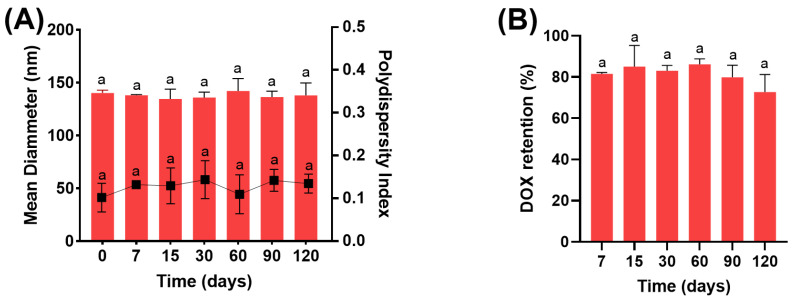
Evaluation of the storage stability of HF-pHSL/EV-DOX. The values of mean diameter, and PDI are represented in (**A**) and DOX retention in (**B**) up to 120 days of storage at 4 °C. Different letters represents statistical differences between the times of storage (*p* < 0.05). The data were tested for homoscedasticity and normality and subsequently analyzed by ANOVA (Tukey’s test).

The hybrid formulation exhibited a mean diameter of 140.2 ± 2.7 nm and a PDI of 0.10 ± 0.03 at day 0, while at day 120, the values were 137.8 ± 11.9 nm and 0.13 ± 0.02, respectively, as shown in Figure 3. Moreover, no changes were observed for the PZ values during 120 days of storage, possibly due to the presence of PEG molecules, which prevent vesicle aggregation by promoting a steric barrier [12]. The DOX retention values for HF-pHSL/EV-DOX were close to 80.0% up to day 90, with a reduction to 72.7 ± 8.5% at day 120. The maintenance of these parameters indicates the stability of the hybrid formulation of liposomes and EVs containing DOX.

### 3.5. Release Study

**Figure 4:**
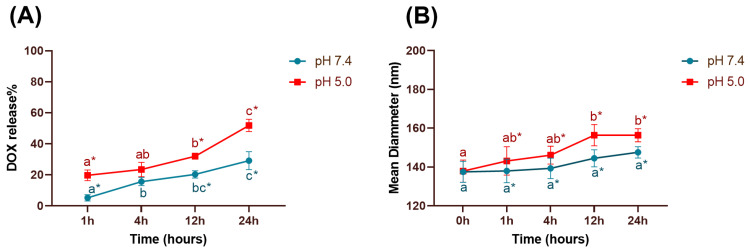
(**A**) Release profile of HF-pHSL/EV-DOX in HBS at pH 5.0 and 7.4. (**B**) Evaluation of the vesicles diameter of HF-pHSL/EV-DOX at pH 5.0 and 7.4. Different letters represent statistical differences between 1 h, 4 h, 12 h, and 24 h at the previous time at the same pH (*p* < 0.05). The asterisk symbol represents statistical differences between pH values 5.0 and 7.4 at the same time (*p* < 0.05). The data were tested for homoscedasticity and normality and subsequently analyzed by ANOVA (Tukey’s test).

As reported in the Section 2, the release of DOX from HF-pHSL/EV-DOX was analyzed at pH 7.4 to mimic physiological release conditions and pH 5.0 to simulate DOX release in the tumor area. The formulation exhibited higher DOX release at pH 5.0 (51.8 ± 3.9%) compared to pH 7.4 (29.2 ± 5.7%), as seen in Figure 4A, suggesting the nanosystem’s pH sensitivity. The hybrid formulation diameter increased significantly at pH 5.0 from 137.9 ± 5.7 nm to 156.3 ± 3.3 nm (Figure 4B), corroborating the indication of pH responsiveness [16].

### 3.6. Cytotoxicity Study

The IC_50_ values, summarized in Table 3, were determined for each treatment to which the cell lines were submitted. The free DOX and the HF-pHSL/EV-DOX treatments achieved similar IC_50_ values, indicating that the drug activity remained after encapsulation in the hybrid nanoformulation for all evaluated human breast cancer cell lines. No significant cytotoxicity was observed after treatment of the three cell lines with blank HF-pHSL/EV, indicating that the cytotoxicity shown by the hybrid formulations is attributed to DOX.

### 3.7. Cellular Uptake Study

**Figure 5:**
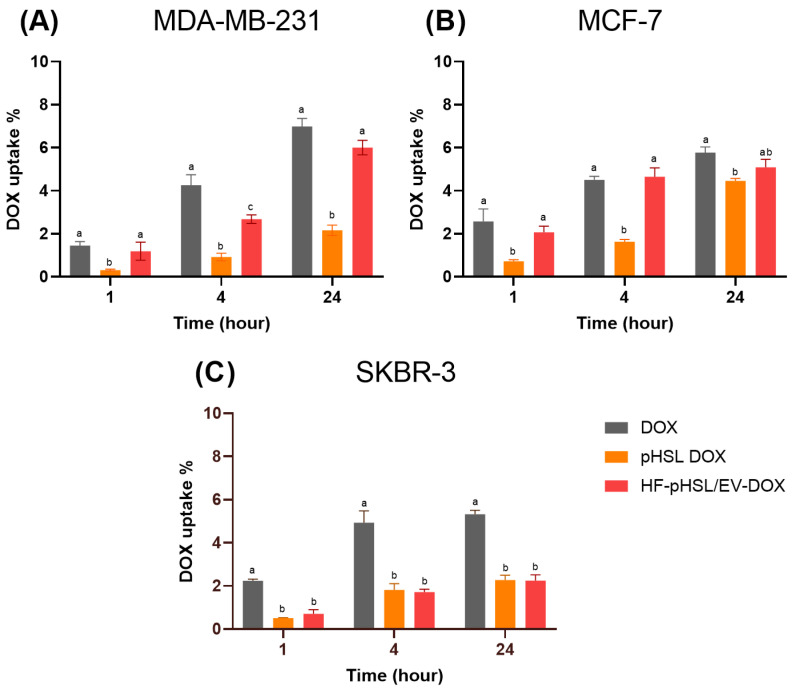
(**A**) Cellular uptake MDA-MB-231, (**B**) MCF-7, and (**C**) SKBR-3 human breast cancer cell lines. Different letters represent statistical differences between 1 h, 4 h, and 24 h (*p* < 0.05).

The cellular uptake of DOX by human breast cancer cell lines MDA-MB-231 (triple negative), MCF7 (ER+/PR+/HER2+), and SKBR3 (HER2+) is shown in Figure 5. The cellular uptake of DOX showed that the internalized DOX amount increased over time for all treatments and across all cell lines. For the MDA-MB-231 cell line, the uptake of DOX after free DOX treatment (6.9 ± 0.4%) and HF-pHSL/EV-DOX treatment (6.0 ± 0.3%) was higher than that of other treatments and similar after 24 h. Similar results were observed for the cellular uptake of DOX after free DOX treatment (5.8 ± 0.3%) and HF-pHSL/EV-DOX treatment (5.1 ± 0.4%) after 24 h using the MCF7 cell line. The SKBR3 breast cancer cell line showed a lower percentage of DOX uptake after treatment with HF-pHSL/EV-DOX (2.3 ± 0.3%), similar to that after treatment with pHSL-DOX (2.3 ± 0.2%) and lower than that after treatment with free DOX (5.3 ± 0.2%).

### 3.8. Migration Test—Wound-Healing Assay

**Figure 6:**
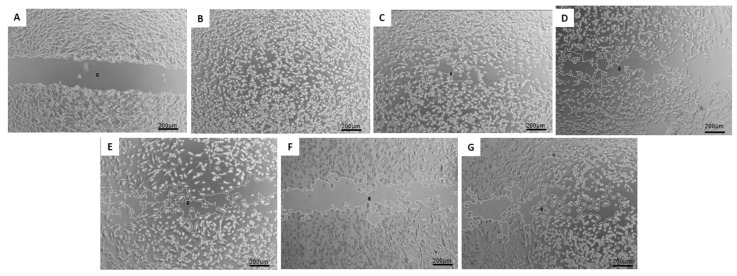
Phase-contrast photomicrography of scratch wounds in MDA-MB-231 breast cancer cell line at (**A**) time zero and after 24 h of exposure to (**B**) DMEM medium supplemented with 1% FBS (control), (**C**) free DOX, (**D**) pHSL-DOX, (**E**) DOXOPEG^®^, (**F**) HF-pHSL/EV-DOX, (**G**) blank HF-pHSL/EV. The images are representative of three independent experiments. Magnification at 5×.

**Figure 7:**
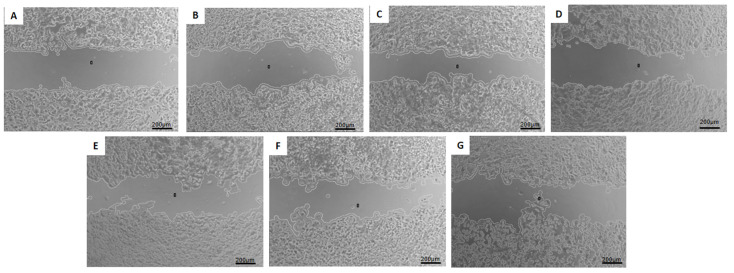
Phase-contrast photomicrography of scratch wounds in MCF-7 breast cancer cell line at (**A**) time zero and after 24 h of exposure to (**B**) MEM medium supplemented with insulin (10 mg/L) and 1% FBS (control), (**C**) free DOX, (**D**) pHSL-DOX, (**E**) DOXOPEG^®^, (**F**) HF-pHSL/EV-DOX, (**G**) blank HF-pHSL/EV. The images are representative of three independent experiments. Magnification at 5×.

The study of cell migration using the wound-healing assay allows for assessing the influence of the analyzed treatments on reducing cellular motility in two-dimensional cultures of confluent cell monolayers [17]. Representative phase-contrast photomicrographs of the scratches and the wound closure after 24 h of exposure to the treatments are shown in Figure 5, Figure 6 and Figure 7.

In the case of MDA-MB-231 cells, treatments with the hybrid nanosystems HF-pHSL/EV-DOX (40.8 ± 5.5%) and blank HF-pHSL/EV (63.2 ± 6.8%) reduced the percentage of cell migration compared to the treatment with free DOX (86.7 ± 6.8%), pHSL-DOX (63.5 ± 9.9%), and the commercial liposomal formulation DOXOPEG^®^ (80.1 ± 4.7%), as shown in Figure 6.

The same pattern of cell migration was observed for the human breast cancer cell line MCF7, as seen in Figure 7. The lowest value of cell migration was obtained with the treatment DOXOPEG^®^ (48.5 ± 9.5%), followed by treatment with HF-pHSL/EV-DOX (51.5 ± 7.2%). Blank hybrid formulation treatment, without DOX encapsulation, could also reduce the percentage of cell migration (56.9 ± 16.8%).

**Figure 8:**
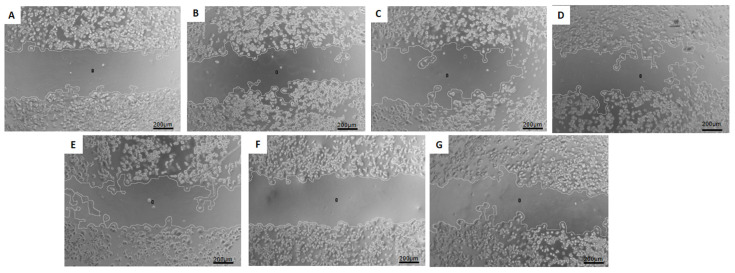
Phase-contrast photomicrography of scratch wounds in SKBR-3 breast cancer cell line at (**A**) time zero and after 24 h of exposure to (**B**) McCoy medium supplemented with 1% FBS (control), (**C**) free DOX, (**D**) pHSL-DOX, (**E**) DOXOPEG^®^, (**F**) HF-pHSL/EV-DOX, (**G**) blank HF-pHSL/EV. The images are representative of three independent experiments. Magnification at 5×.

The human breast cancer cell line SKBR-3 exhibited a migration regarding control similar to that found for the MDA-MB-231 cell line, as shown in Figure 8. The lowest values of cell migration were obtained with the treatments HF-pHSL/EV-DOX (37.7 ± 5.4%), and blank HF-pHSL/EV (54.6 ± 8.5%).

The values for the percentage of relative cell migration compared to the control are described in Table 4. The migration relative to the control was calculated by considering the control cells as having 100% migration, and the migration percentage of each treatment was assessed regarding the control.

### 3.9. Acute Toxicity Study

#### 3.9.1. Evaluation of Animal Mortality and Morbidly

**Figure 9:**
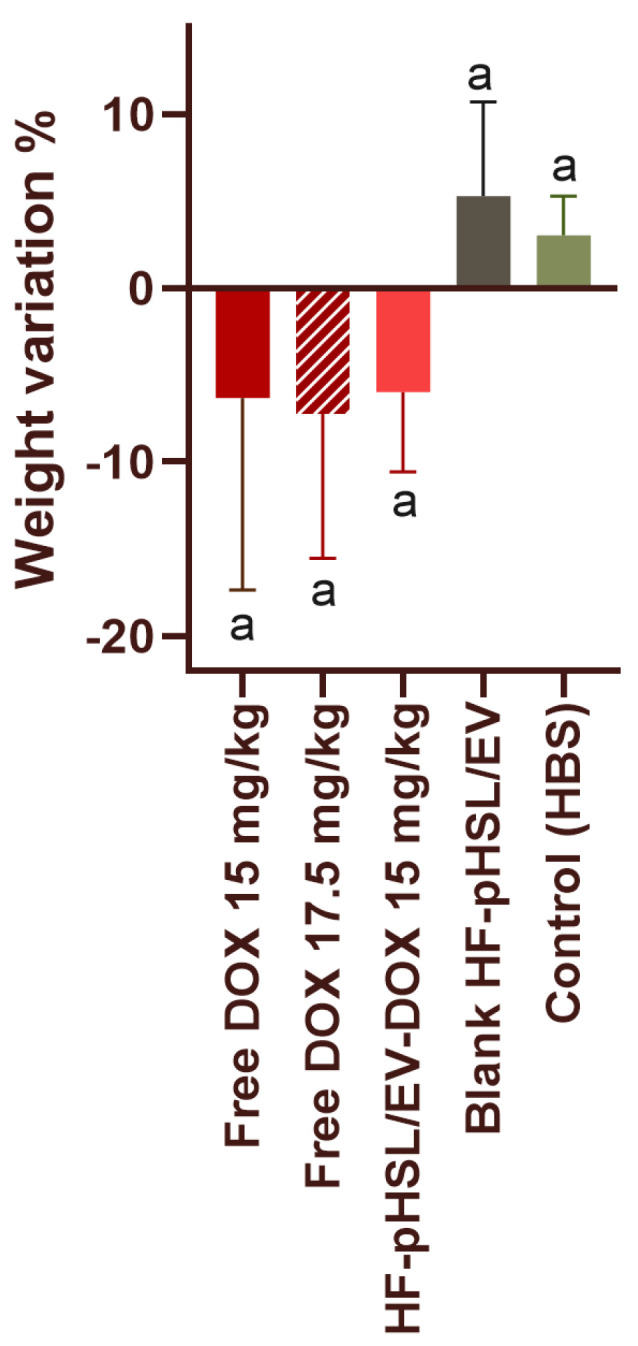
Weight variation in BALB/c healthy female mice after treatment with HBS, free DOX, and HF-pHSL/EV-DOX at different doses. Data are expressed by the mean ± standard deviation of the mean (n = 6, except for the groups treated with free DOX at dose of 15 mg/kg and 17.5 mg/kg, where n = 5). Different letters represent statistical differences between the evaluated parameter (*p* < 0.05). All data were analyzed by one-way ANOVA analysis of variance (Tukey’s test).

The HBS and blank HF-pHSL/EV treatments did not show significant differences regarding mortality and morbidity. These findings indicate the absence of toxicity in these treatments administered to the mice. Both treatment groups did not show substantial weight variation during the monitoring period (3.02 ± 2.26 and 5.26 ± 5.49% in HBS and HF-pHSL/EV treated groups, respectively, as shown in Figure 9), piloerection, or signs of diarrhea.

The assessment of the median lethal dose (LD50) for animals treated with free DOX and HF-pHSL/EV-DOX started with the administration of a dose of 15 mg/kg. Animals treated with free DOX did not show significant signs of toxicity, although they evidenced moderate piloerection and one death occurred on the eighth day of monitoring after administration. The weight of the animals also did not change significantly, with a mean loss of 6.3 ± 11.0% of their weight over the 14 days of study, as shown in Figure 9. Therefore, the next step was administering a 17.5 mg/kg dose. The animals exhibited severe piloerection and signs of diarrhea in the first four days after administration of this single dose. These animals maintained a weight loss of 7.3 ± 8.3% until euthanasia, with one death on the sixth day after administering the single dose. According to the OECD 423 protocol, only one death is insufficient to determine the LD50 [13]. Therefore, the next step was the administration of a 20 mg/kg dose in three more animals. All tested animals from this group died, with two deaths on the seventh day of administration and one on the ninth day. Therefore, the LD50 for treatment with free DOX was estimated to be between 17.5 and 20 mg/kg.

The toxicity study assessed the LD50 for treatment with HF-pHSL/EV-DOX starting with a single dose of 15 mg/kg. The first three animals exhibited piloerection until the fourth monitoring day, with no death. Therefore, the evaluation continued with administration in three additional animals, which confirmed the results of the previous study. For this treatment group, the weight varied by 5.9 ± 4.6%, indicating minimal loss (Figure 9). Then, the next dose of 17.5 mg/kg was administered to three animals. The animals showed intense piloerection, and two deaths on the ninth day of the study. Therefore, according to OECD 423, the assessment of the LD50 for this treatment group concluded with this dose, and the LD50 value was between 15 and 17.5 mg/kg.

#### 3.9.2. Hematological Analysis

The hematological parameters of mice treated with HBS (control), free DOX, HF-pHSL/EV-DOX, and blank HF-pHSL/EV are shown in Table 5. Only one animal was analyzed for the treatment with HF-pHSL/EV-DOX at a dose of 17.5 mg/kg; therefore, statistical analysis was not possible and data are not presented.

The evaluation of white blood cells (WBC) showed a count decrease with the treatment of free DOX at a dose of 17.5 mg/kg and HF-pHSL/EV-DOX at a dose of 15 mg/kg compared to the HBS control group. The other treatments did not show alterations in the count of white blood cells when compared to the HBS control group.

We observed that the number of red blood cells (RBC), and the amounts of hemoglobin (HGB) and hematocrit (HCT) did not show significant differences compared to the HBS control group and among the different treatments. The agranulocytic type of white blood cell series decreased in all treatments compared to the HBS control group. An agranulocyte decrease could indicate a leukopenia process, commonly caused by DOX administration [18,19]. The platelet count (PLT) decreased for the treatment groups with free DOX at a dose of 15 mg/kg and blank HF-pHSL/EV compared to the HBS control group and remained stable for the other treatment groups.

#### 3.9.3. Biochemical Analysis

The biochemical parameters of mice treated with HBS (control), free DOX, HF-pHSL/EV-DOX, and blank HF-pHSL/EV are shown in Table 6. Only one animal was analyzed for the treatment with HF-pHSL/EV-DOX at a dose of 17.5 mg/kg; therefore, statistical analysis was not possible and data are not presented.

Renal function was assessed by measuring creatinine and urea levels, which are plasma metabolites filtered by the kidneys. The results in Table 6 did not show statistical differences between the control and other treatment groups. The same result was observed for cardiac function, assessed by quantifying the creatine kinase MB isoform (CK-MB) activity, mainly found in cardiac muscle. No treatment group showed a statistically significant alteration compared to the HBS control group.

Hepatic function was evaluated by determining the activity of two enzymes: alanine aminotransferase (ALT) and aspartate aminotransferase (AST). An increase in ALT and AST levels in the evaluated plasma of mice treated with HF-pHSL/EV-DOX at a dose of 15 mg/kg was observed compared to the HBS control group, as shown in Table 6. There was also a trend towards an increase in both enzymes for treatments with free DOX (at both doses).

Despite the changes in the biochemical parameters, the extent of damage and its reversibility should be assessed in conjunction with organ histology.

#### 3.9.4. Histopathological Analysis

Histological analyses of the liver, spleen, heart, sternum, and kidneys were performed at the end of the experiment.

**Figure 10:**
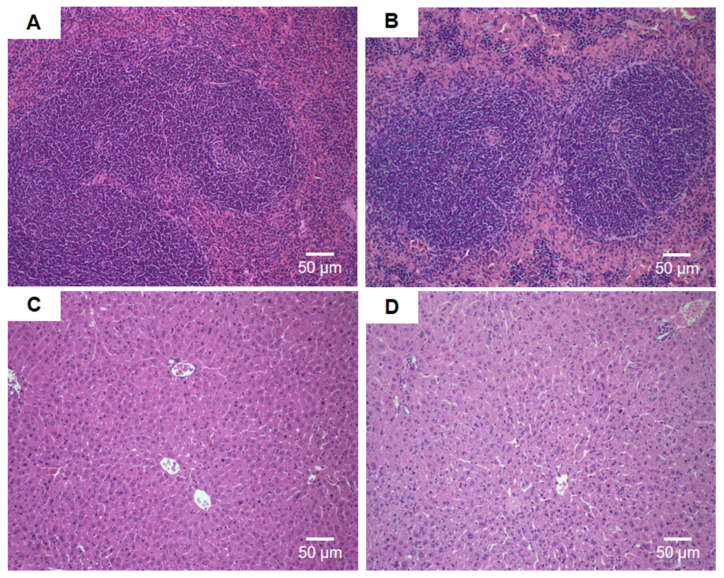
Histological sections of female Balb/c mice spleen (**A**,**B**) and liver (**C**,**D**). (**A**) Represents the groups treated with HBS (control) and blank HF-pHSL/EV. (**B**) Represents the groups treated with free DOX (15 mg/kg and 17.5 mg/kg) and HF-pHSL/EV-DOX at dose of 15 mg/kg. (**C**) Represents the groups treated with HBS (control) and blank HF-pHSL/EV. (**D**) Represents the groups treated with free DOX (15 mg/kg and 17.5 mg/kg) and HF-pHSL/EV-DOX at dose of 15 mg/kg.

The analyses of the spleen did not show histological alterations compared to the control for the groups treated with free DOX (15 mg/kg and 17.5 mg/kg), and HF-pHSL/EV-DOX at a dose of 15 mg/kg, as can be observed in Figure 10A,B. Concerning hepatic toxicity, the groups treated with free DOX at doses of 15 mg/kg, and 17.5 mg/kg, and HF-pHSL/EV-DOX at a dose of 15 mg/kg did not show differences compared to the control group (HBS), as shown in Figure 10C,D.

**Figure 11:**
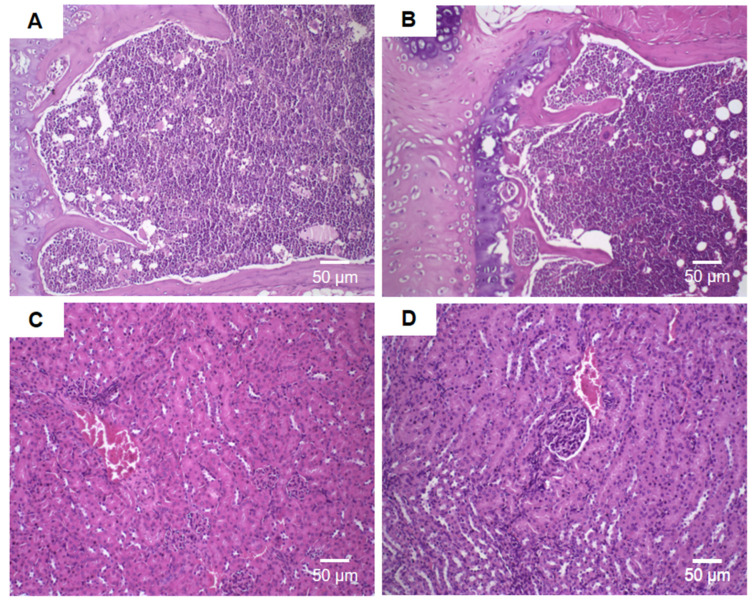
Histological sections of female Balb/c mice sternum (**A**,**B**) and kidney (**C**,**D**). (**A**) Represents the groups treated with HBS (control) and blank HF-pHSL/EV. (**B**) Represents the groups treated with free DOX (15 mg/kg and 17.5 mg/kg) and HF-pHSL/EV-DOX at dose of 15 mg/kg. (**C**) Represents the groups treated with HBS (control) and blank HF-pHSL/EV. (**D**) Represents the groups treated with free DOX (15 mg/kg and 17.5 mg/kg) and HF-pHSL/EV-DOX at dose of 15 mg/kg.

Histological analyses of the sternum (Figure 11A,B) and kidneys (Figure 11C,D) also did not show any histological alteration between the groups treated with free DOX at dose of 15 mg/kg, and 17.5 mg/kg, and HF-pHSL/EV-DOX at a dose of 15 mg/kg compared to the control group (HBS).

**Figure 12:**
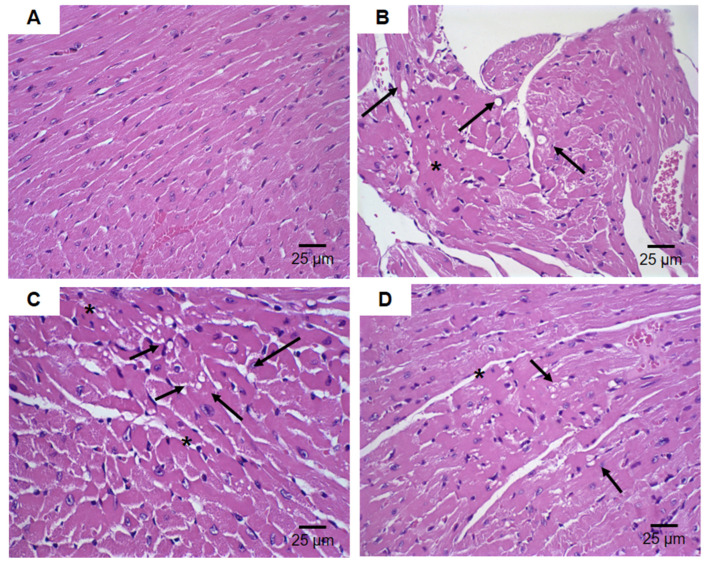
Histological sections of female Balb/c mice heart. (**A**) Represents the groups treated with HBS (control) and blank HF-pHSL/EV. (**B**) Represents the groups treated with free DOX at dose of 15 mg/kg. (**C**) Represents the groups treated with free DOX at dose of 17.5 mg/kg. (**D**) Represents the groups treated with HF-pHSL/EV-DOX at dose of 15 mg/kg. Asterisk indicate areas of hyalinization and arrow areas of vacuolization.

The histological heart sections are shown in Figure 12. The groups treated with free DOX (Figure 12B,C) exhibited more vacuolization of cardiomyocyte areas (indicated by arrows) and areas of hyalinization process (indicated by asterisks), which indicates degenerative processes of muscle fibers. The group treated with HF-pHSL/EV-DOX at a dose of 15 mg/kg showed a focal area of vacuolization of cardiomyocytes, and a hyalinization process area (Figure 12D).

## 4. Discussion

In this study, pegylated pH-sensitive liposomes fused with EVs isolated from human breast cancer cells (MDA-MB-231) were prepared by the film hydration method [10]. Kim and Evers used a similar method, hydrating the lipid film with an EV-containing aqueous solution [20,21]. Another study by Hu et al. promoted the fusion of EVs and liposomes using different methods, such as incubation followed by extrusion [22]. Similarly to these studies, our results proved that the film hydration method could lead to the fusion of EVs and pegylated pH-sensitive liposomes. This fusion was confirmed by total protein quantification using BCA, and the identification of CD63 in our hybrid formulation. The tetraspanin CD63 is a common protein on the surface of EVs and was used as a marker to identify the presence of EVs in the hybrid nanosystem [23,24]. The HF-pHSL/EV-DOX hybrid displayed a monodisperse distribution with a mean diameter of 140.2 ± 2.7 nm, making it suitable for intravenous administration as intended. Liposome size has a significant impact on the in vivo formulation behavior. In cancer applications, it is desirable to have vesicles with a mean diameter near 100 nm to leak and/or be transported by the transendothelial transport from the bloodstream, and for retention in tumor interstitial fluid [25,26]. This nanosystem’s low zeta potential (PZ) value results from DSPE-PEG_2000_ molecules in its lipid composition, which are responsible for reducing the electrophoretic mobility of the nanoparticles. Furthermore, DSPE-PEG_2000_ molecules prevent particle aggregation due to the steric effects of the PEG chains, which justify the excellent storage stability of 90 days for HF-pHSL/EV-DOX [11]. The encapsulation percentage of DOX obtained for the HF-pHSL/EV-DOX formulation was high, with a value of 88.9 ± 2.4%, similar to that obtained in other studies conducted by our research group using the ammonium gradient method [10,11]. The encapsulation method consists of a gradient between HBS outside the nanoparticles and ammonium sulfate in the nanoparticle core. There are two possible mechanisms to explain DOX encapsulation in liposomes using the ammonium sulfate gradient method. As discussed by Ansar and Mudalige (2019), and Haran et al. (1993), the mechanism of encapsulation can be due to the acidification of the intra-liposomal compartment and salting-out effects [27,28]. In the first case, the intra-liposomal (NH_4_)_2_SO_4_ has a low permeability coefficient due to the charge of NH_4_^+^ and SO_4_^2-^ ions. Deprotonated DOX molecules penetrate the lipid bilayer, and once they are inside the hybrid nanosystem, they will be protonated. Thus, ammonia resulting from the reaction between the NH_4_^+^ and –NH_2_ functional group of DOX molecules permeates freely across the liposome membrane to the external medium. Protonated DOX molecules react with SO_4_^2-^ anions, and crystallize as (DOX-NH_3_)_2_SO_4_ [27]. The other mechanism relies on the leakage of the neutral ammonia and protons efflux [28]. The diffusion potential of ammonium ions created is responsible for the influx and accumulation of DOX inside the liposomes [29]. The pH sensitivity of the hybrid formulation was assessed to verify if the incorporation of extracellular vesicles could change the pH-triggered release system. Previous studies carried out by our research group have shown a release of approximately 90% of DOX at pH 5.0 from pH-sensitive liposomes [10,30]. Gomes et al. (2022) showed that a similar hybrid system, consisting of exosomes isolated from the murine breast cancer cell line (4T1) and long-circulating pH-sensitive liposomes, achieved a maximum release of 96.6% at pH 5.0 and 70.1% at pH 7.4. In our study, HF-pHSL/EV-DOX had a release of DOX of 51.8 ± 3.9% at pH 5.0, and 29.2 ± 5.7% at pH 7.4 after 24 h of incubation. Additionally, the vesicle diameter increased significantly at pH 5.0 after 24 h of incubation, while no alterations were observed at pH 7.4. These results suggest that the arrangement of components in the proposed hybrid nanosystem may increase its rigidity, altering its permeation and reducing DOX release at both pH environments.

Breast cancer’s heterogeneous expression of immunohistochemical markers may influence patient treatment and prognosis. Therefore, the in vitro activity evaluation of the hybrid formulation, composed of extracellular vesicles isolated from a triple negative molecular subtype (ER-/PR-/HER2-), was performed on three cell lines with different molecular patterns: MDA-MB-231 (triple negative), MCF7 (ER+/PR+/HER2+), and SKBR3 (HER2+). The results reveal that the hybrid nanosystem containing DOX and EVs from MDA-MB-231 breast cancer cells can inhibit the growth and migration of breast cancer cells with distinct molecular profiles. The study of cell migration using the wound-healing assay enables an assessment of the influence of the analyzed treatments on reducing cellular motility in two-dimensional cultures of confluent cell monolayers, which is relevant to the initial stages of cell migration and invasion in the metastatic process [17]. The wound-healing assay showed free DOX treatment could not inhibit cell migration in treated cells. However, adding EVs in the HF-pHSL/EV-DOX treatment allowed for significant inhibition of wound closure in the three subtypes of breast cancer cell lines. Considering that the metastatic process is a challenge in cancer prognosis, exploring this inhibited cellular motility induced by the association of tumor-isolated EVs and liposomes is crucial. This study emphasizes the potential benefits of this hybrid nanosystem for cancer therapy. This study of cellular DOX uptake also showed that the association of EVs in the lipid bilayer did not prevent DOX uptake by breast cancer cell lines. Kim and Evers [20,21] showed that the EV:liposome particle proportion alters some physicochemical properties and cellular uptake of the bioactive substance encapsulated. Evers showed that the cellular uptake of hybrid formulations was enhanced in the 1:50 EV:liposome proportion compared to the 1:100 EV:liposome proportion [21]. Our hybrid formulation proposed a ratio of approximately 1:300 EV to the liposome. Future studies will be conducted by increasing the EVs/liposome particle ratio to improve cellular uptake of the hybrid nanosystem.

In this study, we also investigated the preliminary acute toxicity of HF-pHSL/EV-DOX. Leukopenia was observed in all groups treated with DOX, with a decrease in the agranulocyte count compared to the HBS treatment group. Similar results were observed in previous studies performed by our research group, where Balb/c mice treated with free DOX, long-circulating and pH-sensitive liposomes (SpHL-DOX), and folate-decorated long-circulating and pH-sensitive liposomes (SpHL-DOX-Fol) evidenced a significant difference in agranulocyte count compared to the saline control [15]. The hepatotoxicity induced by the administration of DOX may be increased when encapsulated in nanoformulations due to its normal liver uptake. When adding human-derived extracellular vesicles to the immunocompetent Balb/c animals, this liver uptake may also increase as a process of antigen elimination, causing an increase in AST and ALT quantification. Further investigation of biodistribution and pharmacokinetics of HF-pHSL/EV-DOX is necessary to evaluate this process. Cardiotoxicity is DOX’s most concerning side effect, and its encapsulation in a hybrid nanosystem reduced the heart damage provoked by the administration of free DOX, as seen in histological analyses [15]. All of these results indicate a perspective of success in the clinical application of HF-pHSL/EV-DOX due to its great cytotoxic activity for different molecular types of breast cancer associated with an excellent toxicity profile.

## 5. Conclusions

The results of this study confirm the successful preparation of the hybrid nanosystem formed by the association of a pegylated and pH-sensitive liposome with extracellular vesicles. The formulation HF-pHSL/EV-DOX exhibits suitable chemical and physicochemical characteristics for intravenous administration, and a good storage stability. Moreover, this hybrid nanosystem showed that the association of EVs in the lipid bilayer does not impair sensitivity to acidic pH, maintaining its ability to respond to changes in the pH of the medium. The cytotoxicity and anti-migratory activity studies showed that HF-pHSL/EV-DOX has a similar cytotoxic effect against breast tumor cell lines with different phenotypic characteristics (triple negative, ER-positive, PR-positive, and HER2-positive), and can prevent cell migration. We should also mention that the blank hybrid nanosystem also has anti-migratory activity. This observation reveals the importance of EVs in hybrid nanosystems for this biological effect. Therefore, these results and the good acute toxicity profile observed show the potential of the hybrid nanosystem in treating breast cancer of different molecular subtypes.

## Figures and Tables

**Table 1:** Chemical and physicochemical characterization of EVs _MDA-MB-231_ and hybrid formulation HF-pHSL/EV-DOX.

Parameter	EVs _MDA-MB-231_	HF-pHSL/EV-DOX
Mean diameter (nm)	181.6 ± 11.5 ^a^	140.2 ± 2.7 ^b^
Polydispersity index	-	0.102 ± 0.033
Zeta potential (mV)	-	−8.2 ± 1.5
Encapsulation of DOX (%)	-	88.9 ± 2.4
Particle concentration (particle/mL)	7.99 ± 0.50 × 10^10 a^	5.01 ± 0.75 × 10^13 b^

The results were presented as mean ± standard deviation of three independent experiments. Different letters indicate statistically significant differences between the evaluated parameters (*p* < 0.05). The data were tested for homoscedasticity and normality and subsequently analyzed by ANOVA (Tukey’s test).

**Table 2:** Quantification of total proteins and positive events for anti-CD63 in EVs, blank pHSL, and blank HF-pHSL/EV samples.

Samples	Protein Concentration (µg/mL)
EVs _MDA-MB-231_	306.2 ± 7.4 ^a^
Blank pHSL	50.2 ± 8.7 ^b^
Blank HF-pHSL/EV	149.3 ± 16.6 ^c^

The results were presented as mean ± standard deviation of three independent experiments. Different letters indicate statistically significant differences between the evaluated parameters (*p* < 0.05). The data were tested for homoscedasticity and normality and subsequently analyzed by ANOVA (Tukey’s test).

**Table 3:** IC_50_ values for DOX, pHSL-DOX, HF-pHSL/EV-DOX, and blank HF-pHSL/EV against different breast cancer cell lines.

Treatment	IC_50_ (µM)		
	MDA-MB-231	MCF-7	SKBR-3
DOX	0.461 ± 0.025 ^a^	0.749 ± 0.137 ^a^*	0.505 ± 0.103 ^a^
pHSL-DOX	0.778 ± 0.161 ^a^	0.974 ± 0.299 ^a^	1.129 ± 0.049 ^a^
HF-pHSL/EV-DOX	0.778 ± 0.161 ^a^	0.537± 0.117 ^a^	0.741 ± 0.167 ^a^
Blank HF-pHSL/EV	>50 ^b^*	8.31 ± 4.8 ^b^*	>10 ^b^*

The results were presented as mean ± standard deviation of three independent experiments. Different letters indicate statistically significant differences between the evaluated parameters within the same cell line (*p* < 0.05). The asterisk symbol indicates a statistically significant difference in the same treatment among different cell lines (*p* < 0.05). The data were tested for homoscedasticity and normality and subsequently analyzed by ANOVA (Tukey’s test).

**Table 4:** Percentage of cell migration in relation to control for MDA-MB-231, MCF-7, and SKBR-3 cell lines evaluated after exposure to the treatments.

Treatment	Wound Healing %		
	MDA-MB-231	MCF-7	SKBR-3
Control (medium + 1% FBS)	100.0 ± 2.7	99.9± 26.1	100.0 ± 16.8 ^bc^
DOX	86.7 ± 6.8 ^abc^	85.4 ± 20.1	104.0 ± 22.7 ^bc^
pHSL-DOX	63.5 ± 9.9 ^ab^	72.4 ± 14.1	129.2 ± 9.7 ^bc^
DOXOPEG^®^	80.1 ± 4.7 ^abc^	48.5 ± 9.5 ^a^	70.9 ± 27.8
HF-pHSL/EV-DOX	40.8 ± 5.5 ^ac^	51.5 ± 7.2 ^a^	37.7 ± 5.4 ^a^
Blank HF-pHSL/EV	63.2 ± 6.8 ^a^	56.9± 16.8 ^a^	54.6 ± 8.5 ^a^

The results were presented as mean ± standard deviation of three independent experiments. “a” indicates statistically significant difference compared to the control treatment (*p* < 0.05). “b” indicates statistically significant difference compared to HF-pHSL/EV-DOX (*p* < 0.05). “c” indicates statistically significant difference compared to blank HF-pHSL/EV (*p* < 0.05). The data were tested for homoscedasticity and normality and subsequently analyzed by ANOVA (Tukey’s test).

**Table 5:** Hematological parameters for healthy Balb/c mice treated with different doses of free DOX, HF-pHSL/EV-DOX, or HF-pHSL/EV.

Blood Components	HBS (Control)	Free DOX		HF-pHSL/EV-DOX	HF-pHSL/EV
		15 mg/kg	17.5 mg/kg	15 mg/kg	
WBC (10^3^/mm^3^)	7.83 ± 2.36	4.14 ± 0.93	3.04 ± 0.86 ^a^	3.05 ± 0.96 ^a^	6.60 ± 3.97
AGRANULOCYTES (10^3^/mm^3^)	5.58 ± 1.52	0.49 ± 0.14 ^a^	0.30 ± 0.16 ^a^	0.57 ± 0.31 ^a^	1.65 ± 0.73 ^a^
GRANULOCYTES (10^3^/mm^3^)	2.00 ± 0.62	3.76 ± 0.68	2.78 ± 0.73	2.42 ± 0.89	3.67 ± 1.76
RBC (10^6^/mm^3^)	7.44 ± 1.68	6.21 ± 1.47	5.64 ± 1.47	5.71 ± 1.78	6.65 ± 1.87
HGB (g/dL)	16.05 ± 4.17	11.92 ± 2.54	10.50 ± 2.42	10.72 ± 3.25	12.90 ± 3.09
HCT (%)	39.06 ± 5.79	31.96 ± 6.60	28.52 ± 6.32	28.88 ± 8.92	33.98 ± 8.99
PLT (10^3^/mm^3^)	612.5 ± 109.9	314.7 ± 153.4 ^ab^	359.0± 116.6	619.0 ± 160.5	244.8 ± 50.7 ^ac^

The results were presented as mean ± standard deviation (n = 6, except for the groups treated with free DOX at 15 mg/kg and 17.5 mg/kg, where n = 5). “a” indicates statistically significant difference compared to the HBS control (*p* < 0.05). “b” indicates statistically significant difference compared to blank HF-pHSL/EV. “c” indicates statistically significant difference compared to the group treated with HF-pHSL/EV-DOX at dose of 15 mg/kg. The data were tested for homoscedasticity and normality and subsequently analyzed by ANOVA (Tukey’s test).

**Table 6:** Biochemical parameters for healthy Balb/c mice treated with different doses of free DOX, HF-pHSL/EV-DOX, or HF-pHSL/EV.

Biochemical Parameters	HBS (Control)	Free DOX		HF-pHSL/EV-DOX	HF-pHSL/EV
		15 mg/kg	17.5 mg/kg	15 mg/kg	
Creatinine (mg/dL)	0.26 ± 0.10	0.35 ± 0.06	0.33 ± 0.06	0.29 ± 0.06	0.34 ± 0.04
Urea (mg/dL)	54.45 ± 10.85	48.40 ± 3.67	48.33 ± 9.44	50.11 ± 9.08	52.94 ± 4.13
ALT (U/L)	30.85 ± 12.95 ^a^	48.37 ± 12.20	42.52 ± 10.85	61.55 ± 5.35	34.05 ± 6.67 ^a^
AST (U/L)	74.73 ± 16.45 ^a^	87.73 ± 11.42	93.24 ± 21.16	124.60 ± 18.98	94.14 ± 22.09
CK-MB (U/L)	25.31 ± 6.37	40.75 ± 23.78	42.54 ± 18.60	39.65 ± 16.83	38.64 ± 13.35

The results were presented as mean ± standard deviation (n = 6, except for the groups treated with free DOX at 15 mg/kg and 17.5 mg/kg, where n = 5). “a” denotes statistically significant difference compared to the HF-pHSL/EV-DOX treatment at dose of 15 mg/kg (*p* < 0.05). The data were tested for homoscedasticity and normality and subsequently analyzed by ANOVA (Tukey’s test).

## Data Availability

The original contributions presented in the study are included in the article/supplementary material, further inquiries can be directed to the corresponding author/s.

## Abbreviations

extracellular vesicles (EVs); doxorubicin (DOX); pegylated pH-sensitive liposome fused with extracellular vesicles encapsulating doxorubicin (HF-pHSL/EV-DOX); pegylated pH-sensitive liposome fused with extracellular vesicles (HF-pHSL/EV); pegylated pH-sensitive liposomes containing DOX (pHSL-DOX).
